# New Perspectives on the Neurobiology of Sign Languages

**DOI:** 10.3389/fcomm.2021.748430

**Published:** 2021-12-13

**Authors:** Karen Emmorey

**Affiliations:** School of Speech, Language and Hearing Sciences, San Diego State University, San Diego, CA, United States

**Keywords:** sign language, fingerspelling, non-manual features, neuroimaging, event-related potentials

## Abstract

The first 40 years of research on the neurobiology of sign languages (1960–2000) established that the same key left hemisphere brain regions support both signed and spoken languages, based primarily on evidence from signers with brain injury and at the end of the 20th century, based on evidence from emerging functional neuroimaging technologies (positron emission tomography and fMRI). Building on this earlier work, this review focuses on what we have learned about the neurobiology of sign languages in the last 15–20 years, what controversies remain unresolved, and directions for future research. Production and comprehension processes are addressed separately in order to capture whether and how output and input differences between sign and speech impact the neural substrates supporting language. In addition, the review includes aspects of language that are unique to sign languages, such as pervasive lexical iconicity, fingerspelling, linguistic facial expressions, and depictive classifier constructions. Summary sketches of the neural networks supporting sign language production and comprehension are provided with the hope that these will inspire future research as we begin to develop a more complete neurobiological model of sign language processing.

## INTRODUCTION

Once sign languages were shown to be natural, full-fledged languages, it became clear that investigating their neural substrates could provide unique evidence for how the brain is organized for human language processing. Beginning in the 1980’s, case studies of deaf signers with unilateral left hemisphere damage revealed impairments in sign language production and comprehension; in contrast, deaf signers with right hemisphere damage did not exhibit aphasic deficits (Poizner et al., 1987). In addition, as with spoken languages, left frontal damage was found to result in sign production deficits (Hickok et al., 1996), while left temporal lobe damage resulted in sign comprehension deficits (Hickok et al., 2002). Early neuroimaging studies of sign production (Petitto et al., 2000) and comprehension (Neville et al., 1998) supported these findings in neurotypical signers, revealing activation in the left inferior frontal gyrus for sign production and in left superior temporal cortex for sign comprehension. This evidence showed that the same key frontal and temporal regions in the left hemisphere support both spoken and signed languages; for reviews of these earlier studies see Corina and Knapp (2006), Emmorey (2002), MacSweeney et al. (2008a). Further, early lesion and neuroimaging studies indicated that the right hemisphere is involved in processing classifier/depictive constructions, particularly those constructions that express spatial relationships (e.g., Emmorey et al., 2002; Atkinson et al., 2005). This work suggested that the right hemisphere may be uniquely involved in processing some of the visual-spatial aspects of sign language structure.

This article builds on this early work and focuses primarily on what we have learned about the neural bases of sign language processing within the last ~15 years. The aim is to sketch a neurobiological model of sign language production and comprehension that includes linguistic phenomena that are fundamentally shaped by the visual-manual modality (pervasive iconicity, depictive classifier constructions, and the modality overlap with manual actions) and variables that are unique to sign languages (e.g., visual-manual phonological units, mouthing, fingerspelling, the use of signing space for co-reference). Production and comprehension processes are addressed separately in order to capture whether and how output and input differences between sign and speech might impact the neural substrates supporting linguistic articulation versus perception. By widening our scientific lens in this way, we move beyond classic models of brain-language relationships which focus on shared neural substrates for signed and spoken languages, and we can begin to develop richer models that map psycholinguistic processes and linguistic units that may or may not be shared with spoken language onto a functional neuroanatomical network that supports sign language processing.

## THE NEUROBIOLOGY OF SIGN LANGUAGE PRODUCTION

Psycholinguistic evidence indicates that production processes are largely parallel for speech and sign. For example, both sign and speech production require phonological assembly of sublexical units (handshape, location, and movement for sign language), as evidenced by systematic production errors (slips of the hand: Hohenberger et al., 2002; slips of the tongue: Fromkin, 1971). Both sign and speech production involve a two-stage process in which lexical semantic representations are retrieved independently of phonological representations, as evidenced by tip-of-the-tongue and tip-of-the-finger states (Brown and McNeill, 1966; Thompson et al., 2005). At the sentence level, syntactic priming in sentence production occurs for both signed and spoken languages (Bock, 1986; Hall et al., 2015), supporting a distinction between syntactic and conceptual representations. In this section, we explore the neural regions that are involved at these processing levels, starting with sign articulation and phonological encoding and then discussing regions that are associated with higher-level processing: lexical retrieval (including fingerspelled words) and sentence and phrase production (including classifier constructions). We end this section with a summary sketch of the neural network for sign production.

### Sign Articulation and Phonological Encoding

Although there are detailed models of the neural underpinnings of speech articulation (e.g., Tourville and Guenther, 2011), the data needed to develop such a model for sign articulation are relatively sparse. Nonetheless, recent research has begun to illuminate the neural circuits that are recruited during signing. One obvious difference between sign and speech production is the nature of the linguistic articulators. The manual articulators for sign are relatively large, can move independently within a large space, and are visible to the addressee but not always to the signer (signing is not visually guided). These sensorimotor characteristics are likely to impact the neural networks for language production in the visual-manual modality.

To begin to identify neural regions involved in the manual articulation of signs, Emmorey et al. (2016) used positron emission tomography (PET) and an English translation task to elicit different sign types in American Sign Language (ASL): one-handed signs, two-handed signs, and body-anchored (i.e., body contact) signs. In the baseline comparison task, deaf ASL signers also viewed English words and indicated whether the word contained a descending letter (e.g., j, p) using the signs YES or NO. The translation task required retrieval of an ASL sign, phonological assembly, and different articulation demands depending on sign type, while the baseline comparison task did not involve these processes. Not surprisingly, the production of two-handed signs engaged sensorimotor cortex in both hemispheres, while one-handed signs activated sensorimotor cortex in the left hemisphere (all signers were right-handed). Less activation in parts of the motor circuit was found for two-handed compared to one-handed signs, possibly because handshape and movement goals could be spread across the two limbs for symmetrical two-handed signs. Within non-linguistic motor domains, cortical activity in premotor regions is reduced when the goal of finger movements (e.g., to direct a cursor) is spread across the two hands, rather than controlled by a single hand (Post et al., 2007). In addition, the articulation of one-handed signs may require active suppression of the non-dominant hand (cf. Cincotta and Ziemann, 2008). Thus, the production of two-handed symmetrical signs may require fewer neural resources than the production of one-handed signs.

Emmorey et al. (2016) also found that the production of body-anchored (one-handed) signs engaged the left superior parietal lobule (SPL) to a greater extent than one-handed signs produced in neutral space. Emmorey et al. (2016) hypothesized that increased SPL activation reflects the increased motor control and somatosensory monitoring needed to direct the hand to a specific location on the body. For nonlinguistic motor tasks, SPL is known to be involved in planning reaching movements and updating postural representations of the arm and hand when movements are not visually guided, as in signing (Parkinson et al., 2010; Striemer et al., 2011). In addition, data from two electrocorticography (ECoG) studies with hearing bimodal bilinguals provide evidence that sign, but not speech production activates left superior parietal cortex (Crone et al., 2001; Shum et al., 2020; see also Emmorey et al. (2007) and Emmorey et al. (2014) for PET evidence). Shum et al. (2020) found that activity in left SPL immediately preceded sign production (~120 ms prior to initiating hand movement), suggesting that left SPL plays an important role in planning sign articulation. Furthermore, this temporal pattern of left SPL activity was not observed for non-linguistic reaching movements or for speech production. In addition, Crone et al. (2001) reported that electrical cortical stimulation of regions in left SPL interfered with sign (but not speech) production, although the nature of this interference was not specified. Overall, the data indicate that left SPL is uniquely involved in the planning and execution of signs, but not spoken words.

The supramarginal gyrus (SMG) is another region known to be involved in sign language production. Corina et al. (1999a) was the first to suggest that left SMG was involved in phonological encoding of signs based on cortical stimulation mapping in a deaf ASL signer undergoing awake craniotomy for surgical treatment of epilepsy. Stimulation of left SMG resulted in phonological errors (e.g., handshape substitutions), rather than articulatory execution errors (e.g., lax articulation of an intended sign). In the Emmorey et al. (2016) PET study, the conjunction analysis revealed that all sign types activated the SMG bilaterally, with more extensive activation in the left hemisphere. Activation in left SMG has also been found when signers name pictures/videos in ASL (Emmorey et al., 2003; Kassubek et al., 2004; Okada et al., 2016) or Chinese Sign Language (CSL) (Hu et al., 2011). Further, activation in left SMG is observed during covert production when signers are asked to “sub-manually” name pictures (Kassubek et al., 2004), mentally rehearse learned pseudosigns (Buchsbaum et al., 2005) or make phonological decisions about internally-generated signs using picture stimuli (i.e., do the British Sign Language (BSL) sign names have the same location?) (MacSweeney et al., 2008b). Thus, the SMG is engaged when signers retrieve or rehearse the form of signs, even when overt articulation does not occur. The SMG is also engaged during speech production (Hickok, 2012), and it is possible that this region supports amodal processes such as retrieval of phonological lexical forms or phonological computations. However, direct contrasts between speech and sign production consistently reveal greater activation in SMG (and SPL) for sign production (Braun et al., 2001; Kassubek et al., 2004; Emmorey et al., 2007, Emmorey et al., 2014). Thus, left SMG may be more extensively involved in phonological processing for sign compared to speech production. Another possibility is that some regions within the SMG are involved in modality-specific processing and representation of sign phonology (possibly more dorsal regions; see Buchsbaum et al., 2005), while other regions support amodal phonological functions (perhaps more anterior regions that overlap with speech production).

Finally, the recent ECoG study by Leonard et al. (2020) found that single electrodes over SMG, pre-central (motor) cortex, and post-central (sensory) cortex in the left hemisphere exhibited neural selectivity for specific ASL handshapes and/or locations. In this study, a profoundly deaf signer with early ASL exposure produced ASL signs while viewing real signs and pseudosigns as part of a lexical decision paradigm. However, rather than performing the “yes/no” lexical decision task, the participant often repeated the ASL sign, fingerspelled the sign, or repaired the pseudosign to a real sign. The finding of neural selectivity for phonological units in ASL within sensorimotor cortex is parallel to what has been found for speakers. For example, using ECoG data Bouchard et al. (2013) identified speech-articulator representations (such as the tongue and lips) that were laid out somatotopically along sensorimotor cortex, and spatiotemporal patterns of neural activity that were hierarchically organized by articulatory-defined phonetic features, such as lip-rounding or tongue position. Similarly, Leonard et al. (2020) found that the spatial distribution of the neural activity across location- and handshape-selective electrodes was clustered along a linguistically-relevant hierarchy, e.g., open and closed handshapes were clustered together at a low (phonological) level, while fingerspelled words and lexical signs were clustered together at a higher (lexical) level. Further, these cortical responses were specific to linguistic production, rather than simply reflecting general motor actions of the hand and arm because these cortical patterns were not observed during transitional movements. This study presents some of the first evidence that sublexical phonological representations are supported by the same neural principles and hierarchical architecture, regardless of language modality.

### Lexical Production

Lexical sign production has been found to be more strongly left-lateralized than spoken word production (Gutierrez-Sigut et al., 2015; Gutierrez-Sigut et al., 2016). Gutiérrez-Sigut and colleagues used functional transcranial Doppler sonography (fTCD) to investigate hemispheric lateralization during speech and sign production in neurotypical adults. fTCD measures event-related changes in blood flow velocity within the middle cerebral arteries in the two hemispheres. Hearing BSL-English bilinguals exhibited stronger left lateralization for sign than speech production when performing verbal fluency tasks (e.g., produce as many animal signs/words as you can in a short time period). A control experiment with sign-naïve participants indicated that the difference in degree of laterality was not driven by greater motoric demands for manual articulation. Left-lateralization was stronger for overt than covert sign production in deaf BSL signers, but the strength of lateralization was not correlated with the amount of time moving the right hand during overt signing, indicating that strong left-lateralization is not simply due to right-hand motor demands. In addition, covert sign production was more strongly left-lateralized than overt word production in hearing speakers. Gutierrez-Sigut and colleagues speculated that greater left lateralization for sign production compared to word production might be due to increased use of somatosensory self-monitoring mechanisms and/or to the nature of phonological encoding for signs.

Left inferior frontal cortex (IFC) is one region that is consistently engaged during both single sign and single word production, and damage to left IFC results in impairments in lexical production. This region is associated with several linguistic functions in spoken languages, with more anterior regions (Brodmann Area (BA) 47) associated with lexical-semantic processes and more posterior regions associated with phonological processing (BA 45) (Devlin et al., 2003). Corina et al. (2003) reported nearly identical activation in left IFC (BA 45, 47) when ASL signers performed a verb generation task with either their right or left hand, supporting the hypothesis that this region supports lexical processing and demonstrating that left IFC activation is not driven by right-handed signing. Further evidence that left IFC is involved in lexical retrieval or selection processes is that this region was more engaged when signers translated from an English word to an ASL sign than when they fingerspelled a printed English word or detected a descending letter in a word–the latter two tasks do not require lexical retrieval or selection of an ASL sign (Emmorey et al., 2016). In addition, left IFG was engaged when deaf and hearing signers imitated CSL signs, but activity in left IFC was not observed for non-signers who did not know the meanings of the signs (Li et al., 2014), again pointing to a role for left IFC in lexical semantic and/or phonological processing during lexical production.

With respect to the connectivity of the neural network for lexical sign production, evidence from cortical stimulation in another deaf signer suggests that the posterior, superior region of the left inferior frontal gyrus (IFG) is connected both functionally and anatomically to the superior part of the left SMG (Metellus et al., 2017). Stimulation of this left IFG region elicited sign production errors (mistakes in handshape or location or sign blockage) in both object-naming and word-translation tasks. Similar errors were observed when the arcuate fasciculus (the fiber tract connecting the IFG and SMG) was stimulated. Further, stimulation of this left IFG region induced an after-discharge (stimulation-induced neural spiking) that occurred 6–8 s later in left superior SMG, providing evidence for functional connectivity between these regions. Other cortical stimulation studies with deaf ASL signers have found that stimulation of left SMG can produce lexical errors (Corina et al., 1999a; Leonard et al., 2020). In addition, most studies of lexical sign production report neural activity in left IFG and SMG (ASL: Okada et al., 2016; San José-Robertson et al., 2004; CSL: Hu et al., 2011; Li et al., 2014). Together, these data provide evidence for a dorsal fronto-parietal network that supports lexical sign production.

Finally, studies that elicit sign (and word) production using picture-naming tasks typically report neural activity in left inferior temporal (IT) cortex (Damasio et al., 1996; Emmorey et al., 2003, Emmorey et al., 2007). This neural region is hypothesized to mediate between conceptual representations of objects and lexical retrieval processes. In addition, there appears to be a topographic gradient along left IT, such that unique object concepts (e.g., known people, landmark buildings) are represented in the anterior temporal pole, while more general object concepts (e.g., animals, tools) are represented along posterior IT (Grabowski et al., 2001; Martin and Chao, 2001). Evidence for such a semantic gradient has also been found for sign language (Emmorey et al., 2003; Emmorey et al., 2013). In addition, the left posterior middle temporal gyrus (pMTG) is also often engaged during sign production elicited by picture/video naming, translation tasks, and verb generation tasks (CSL: Hu et al., 2011; ASL: San José Roberson et al., 2004; Okada et al., 2016). Activation in this region likely reflects an interface that links lexical semantic representations to phonological representations (Indefrey and Levelt, 2004; Hickok and Poeppel, 2007).

#### Iconicity and Lexical Retrieval

The traditional view has been that iconicity (i.e., the perceived resemblance between a form and its meaning) plays no role in sign language acquisition or processing (Emmorey, 2002; Meier and Newport, 1990). However, new evidence is emerging that iconicity can facilitate first language acquisition (BSL: Thompson et al., 2012; ASL: Caselli and Pyers, 2020) and impact sign processing (Thompson et al., 2009; Vinson et al., 2015; Navarrete et al., 2020; Occhino et al., 2020). Further, iconicity is much more pervasive in sign languages, possibly due to the greater ability of the body (vs the vocal tract) to depict actions and objects. These facts raise several questions. Does the more “embodied” nature of iconic signs impact their neural representation? Do iconic signs exhibit different functional connectivity within the brain (e.g., with greater connectivity to sensorimotor cortex)? Is there a neurophysiological response that is related to lexical iconicity, as found for lexical frequency and concreteness in spoken language (Kutas and Federmeier, 2011)?

Thus far, the evidence that iconicity impacts the neural network supporting lexical retrieval and sign production is mixed. In a PET study, Emmorey et al. (2004) reported no significant differences in neural activity when deaf ASL signers named pictures of actions with iconic handling signs (e.g., **STIR**, **BRUSH-HAIR**) versus less iconic, non-handling verbs (e.g., **YELL**, **READ**). Similarly, Emmorey et al. (2011) reported no significant differences between iconic “pantomimic” verbs (e.g., **HAMMER**, **SCRUB**) and non-iconic verbs (e.g., **SWEEP**, **MEASURE**) when ASL signers generated verbs associated with objects. In both PET studies, sign production engaged left IFG (compared to a baseline task), but this neural activity was not modulated by iconicity.

In contrast to these neuroimaging studies, results from event-related potential (ERP) studies suggest that iconicity can modulate the brain response during lexical production (Baus and Costa, 2015; McGarry et al., 2021a; Gimeno-Martinez and Baus, 2021). Behavioral results from picture-naming studies across different sign languages have found that iconic signs are retrieved more quickly than non-iconic signs: Catalan Sign Language (LSC; Baus and Costa, 2015), Italian Sign Language (LIS, Navarrete et al., 2017; Peressotti et al., 2018), BSL (Vinson et al., 2015), and ASL (Sehyr and Emmorey, Forthcoming). For these studies, iconicity is typically assessed using a rating scale with deaf signers or hearing non-signers rating the degree to which a sign form resembles its meaning (ratings by the two groups are highly correlated; Sehyr and Emmorey, 2019). Baus and Costa. (2015) found an early ERP effect (70–140 ms after picture onset) when hearing signers named pictures in LSC, which they suggested reflects early engagement of the conceptual system, with greater activation of semantic features for iconic signs. McGarry et al. (2021a) found that iconicity modulated the N400 response, with a larger N400 for iconic than non-iconic signs, when deaf ASL signers named pictures. McGarry et al. (2021a) hypothesized that this effect was similar to a concreteness effect. Concrete words elicit a larger N400 than abstract words, which is generally attributed to increased activation of perceptual and action-related semantic features associated with concrete words (e.g., Barber et al., 2013). The concreteness-like N400 response for iconic sign production could reflect more robust encoding of sensorimotor semantic features that are depicted by these signs and that are emphasized by the picture naming task.

In fact, Gimeno-Martinez and Baus (2021) provide evidence suggesting that ERP effects of iconicity may be task-dependent because a larger negativity for iconic signs was only observed for LSC picture-naming, but not for a Spanish-LSC translation task. One possible explanation for this finding is that the structural mapping between the visual features of pictures and the visual form of iconic signs (e.g., the ASL sign BIRD depicts a bird’s beak and maps to the beak of a bird in a picture) facilitates lexical retrieval and leads to increased semantic feature activation (and a larger N400 for iconic signs). Another possibility is that the translation task requires little semantic processing (Navarrete et al., 2015) and therefore iconicity does not impact the neural response for this task. However, preliminary results from McGarry et al. (2021b) show effects of iconicity (larger negativity for iconic signs) in an English-ASL translation task, perhaps due to more semantic mediation for this group of signers. This finding supports the hypothesis that iconic signs have a more robust representation of sensorimotor semantic features than non-iconic signs. Overall, however, these mixed results indicate that more work is needed to establish whether or not iconic signs have a distinct neural representation and the extent to which iconicity effects are task-dependent (and why).

#### Production of Fingerspelled Words

Fingerspelling systems that represent the alphabetic orthography of the surrounding spoken language exist for a number of sign languages and can be one-handed (as in ASL) or two-handed (as in BSL). Fingerspelled words differ from lexical signs because they require the production of sequences of hand configurations in neutral space without movements to the body (except to the non-dominant hand for two-handed systems). Thus, the articulatory demands differ for fingerspelled words and lexical signs. Emmorey et al. (2016) found that when deaf ASL signers fingerspelled in response to written English words, ipsilateral (right) motor cortex was recruited in addition to left motor cortex, which was somewhat surprising because fingerspelling was produced by the dominant right hand. However, research with non-linguistic hand actions indicates that ipsilateral motor responses increase with the complexity of hand movements (Verstynen et al., 2005). Emmorey et al. (2016) hypothesized that right motor cortex may contribute to fine motor control of right-handed fingerspelling via callosal connections to the left hemisphere or via uncrossed descending projections (or both). ASL fingerspelling also recruited the cerebellum to a greater extent than the production of one-handed signs, which likely reflects the role of the cerebellum in the precise timing needed to rapidly articulate a string of complex handshapes (the average length of fingerspelled words in this study was six handshapes).

Compared to one-handed signs, Emmorey et al. (2016) found that the production of fingerspelled words engaged the left fusiform gyrus, encompassing the visual word form area (VWFA), a region involved in orthographic processing of words and letters. Activation in the VWFA in this study likely reflects the more detailed orthographic analysis required to fingerspell a written word presented on the screen than to translate a written word into ASL. Interestingly, however, when ASL signers named pictures of famous people using fingerspelling, activation in the fusiform gyrus including the VWFA was also observed (in contrast to a control face-orientation decision task; Emmorey et al., 2003), and VWFA activation was not found for hearing speakers who named the same people with spoken English (Damasio et al., 1996). This result suggests that the production of fingerspelling recruits the VWFA, even without a written prompt, and demonstrates that the function of the VWFA is not limited to the orthographic representation of printed text.

### Sentence and Phrase Production

Very few lesion or neuroimaging studies have examined the neural basis of phrase or sentence production in sign languages. Poizner et al. (1987) described an ASL signer with a large left frontal lesion (GD) who produced simplified (“telegraphic”) sentences. However, damage that is circumscribed to Broca’s area (BA 45/44) does not appear to result in simplified or ungrammatical sentence production, but does result in persistent phonological errors (Hickok et al., 1996). Poizner et al. (1987) described another aphasic ASL signer (PD) who produced fluent signing but with grammatical errors (e.g., incorrect aspectual morphology). PD also failed to maintain spatial agreement across sentences, i.e., he failed to preserve a consistent association between a referent and a location in signing space. PD’s lesion involved a subcortical region underneath Broca’s area, extending into white matter underlying the inferior parietal lobule (SMG and the angular gyrus). Similarly, an aphasic ASL signer (WL) described by Corina et al. (1992) had damage to Broca’s area and underlying white matter tracts, as well as damage to white matter underlying the inferior parietal lobule and a small lesion in left SMG. WL’s sentence production was also fluent (with frequent phonological errors), but his sentences consisted largely of uninflected verbs with few nouns, and virtually no pronouns. Another aphasic signer (LK) first described by Chiarello et al. (1982) had a lesion in left parietal cortex (including SMG and angular gyrus) which spared the inferior frontal gyrus and superior temporal cortex. LK produced fluent signing (with many phonological errors), but she used pronouns inconsistently and failed to establish locations for referents in her spontaneous signing (see also Poizner et al., 1987). The sentence production of “Charles,” a BSL aphasic signer, was severely impaired - his description of the Cookie Theft picture was composed almost entirely of gesture (Marshall et al., 2004). Interestingly, his lesion was located in the left posterior frontal and parietal lobes with only possible involvement of the temporal lobe, suggesting a weaker role for the posterior temporal cortex in sign sentence production (compared to sentence comprehension, see below). These sparse lesion data do not provide many clues to the neural substrates that support sentence-level computational processes, beyond lateralization to the left hemisphere (i.e., these types of errors do not occur with right hemisphere damage). Nonetheless, the case studies of PD, WL, and LK suggest that left inferior parietal cortex (SMG and the angular gyrus) may be involved in pronoun use and reference establishment in signing space. All three had damage involving parietal cortex and exhibited specific impairments in the use of pronouns or agreeing verbs.

With respect to neuroimaging data, there is only one study that targeted phrase-level sign production. Blanco-Elorrieta et al. (2018) used magnetoencephalography (MEG) to investigate the neural circuits that support online construction of linguistic phrases in both sign (ASL) and speech (English). Two-word compositional phrases and two-word non-compositional “lists” were elicited from deaf signers and hearing speakers using identical pictures. In one condition, participants combined an adjective and a noun to describe the color of the object in the picture (e.g., white lamp) and in the control condition, participants named the color of the picture background and then the object (e.g., white, lamp). For both signers and speakers, phrase building activated the left anterior temporal lobe (LATL) and ventromedial prefrontal cortex (vmPFC) more than the list condition, and with a similar time course. Neural activity related to combinatorial processing began in vmPFC at about 100–150 ms, followed by an increase in activity in LATL. The vmPFC is hypothesized to be involved in constructing combinatorial plans (Pylkkänen et al., 2014), while the LATL is hypothesized to be involved in computing the intersection of conceptual semantic features (Poortman and Pylkkänen, 2016). Very similar effects for ASL and English were confirmed by a representational similarity analysis (RSA). Thus, the phrasal combinatory processes supported by vmPFC and LATL are likely modality-independent.

#### Production of Classifier Constructions

Classifier constructions, also known as depictive constructions, are found in most, if not all sign languages and are complex expressions that convey information about the relative location, path and manner of movement, and the size and shape of a referent (see papers in Emmorey, 2003). Damage to either the left or the right hemisphere can cause impairments in the production of these constructions. Hickok et al. (2009) found that right hemisphere damaged ASL signers (*n* = 8) produced significantly more errors on classifier signs than lexical signs in a narrative picture description task, while left hemisphere damaged signers (*n* = 13) produced a similar number of errors for both types of signs. Neuroimaging research has confirmed that the right hemisphere (specifically, right parietal cortex) is engaged during the production of classifier constructions that express spatial relationships (Emmorey et al., 2002, Emmorey et al., 2005, Emmorey et al., 2013).

The PET study by Emmorey et al. (2013) was designed to tease apart the neural regions that support the retrieval of entity classifier handshapes that express object type (e.g., cylindrical object, long-thin object, vehicle, etc.) and the regions that support the expression of location and movement path by where the hands are moved or placed in signing space. Deaf ASL signers performed a picture description task in which they named objects or produced classifier constructions that varied in location, movement, or type of object. In contrast to the gradient, analog expressions of location and motion, the production of both lexical signs and object classifier handshapes engaged left IFG and left inferior temporal cortex, supporting the hypothesis that classifier handshapes are categorical morphemes that are retrieved via left hemisphere language regions. Classifier constructions expressing locations or movement paths both engaged posterior SPL bilaterally. One potential explanation for this result is that right SPL is activated due to the need to mentally transform the visual representation of object locations and movements shown in the picture into a body-centered reference frame for sign production (cf. Harris and Miniussi, 2003), while left SPL is activated due to the need to reach toward target locations in signing space for these constructions (Emmorey et al., 2016). In addition, the left intraparietal sulcus was more engaged when producing location than movement constructions, similar to the results for comprehension (see *Comprehension of Classifier Constructions*; McCullough et al., 2012). Overall, these results indicate that classifier handshapes, like lexical signs, are represented and retrieved via a left fronto-temporal network, while the analog, depictive expression of location and movement within these constructions is supported by bilateral superior parietal cortex.

### Sign Language Production: Summary

**FIGURE 1 |:**
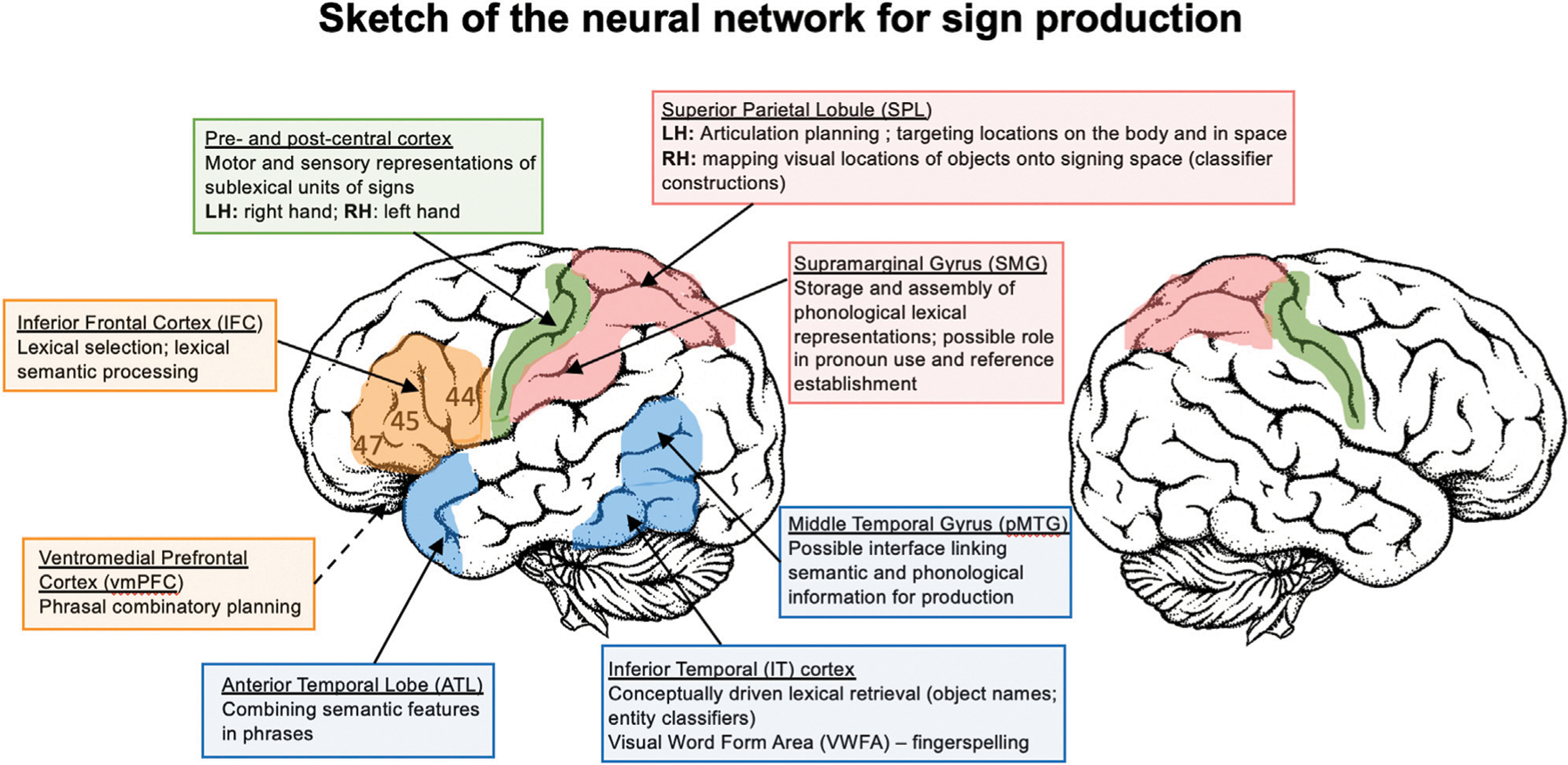
A sketch of the neural network that supports the production of sign language. LH = left hemisphere; RH = right hemisphere.

Sign production is strongly left-lateralized, even more so than speech (Gutierrez-Sigut et al., 2015; Gutierrez-Sigut et al., 2016). Figure 1 provides a sketch of the left hemisphere neural network for sign language production, based on the studies reviewed here. The evidence thus far indicates that phonological encoding for signs involves left parietal cortex. Left SPL is recruited during the planning of sign articulation, (Shum et al., 2020) as well as in monitoring and guiding the hand toward locations on the body (Emmorey et al., 2016), and left SMG appears to be engaged in the storage and assembly of phonological units (Buchsbaum et al., 2005; Corina et al., 1999a). Bimanual coordination for two-handed signs does not necessitate increased involvement of the motor circuit; rather, production of these signs requires fewer neural resources, possibly because production goals can be spread across the two articulators (at least for symmetrical signs) (Emmorey et al., 2016). Sensorimotor cortex exhibits selectivity to linguistically contrastive hand configurations and body locations in a manner that is parallel to the speech articulators, but of course this selectivity occurs within different regions along sensorimotor cortex (Leonard et al., 2020). Finally, in contrast to comprehension (see *Perception of Non-Manual Features*), almost nothing is known about the neural substrate that supports the production of non-manual sublexical components of sign (e.g., mouth gestures, linguistic facial expressions), beyond lateralization to the left hemisphere (Corina et al., 1999b).

The retrieval of lexical signs engages a fronto-parietal network consisting of left IFC and SMG. More anterior regions of left IFC (BA 45, 47) are likely involved in lexical selection and semantic processes, while the SMG is likely involved in phonological processing (Corina et al., 2003; Emmorey et al., 2016). Left pMTG is hypothesized to link lexical semantic representations with phonological representations (Indefrey and Levelt, 2004; Hickok and Poeppel, 2007). For picture naming, left IT is hypothesized to mediate between conceptual processing of objects and lexical retrieval (Emmorey et al., 2003). The retrieval of iconic signs may more robustly activate sensorimotor semantic networks compared to non-iconic signs (Navarrete et al., 2017; McGarry et al., 2021a), but more research is needed. The production of fingerspelled words engages the visual word form area, suggesting a neural link between fingerspelling and orthographic representations (Emmorey et al., 2003; Emmorey et al., 2016). The production of location and motion classifier constructions is supported by bilateral superior parietal cortex (Emmorey et al., 2002; Emmorey et al., 2005; Emmorey et al., 2013). The data suggest that activation in left SPL may be associated with the need to target locations in signing space, while right SPL may be engaged in mapping visual representations of figure and ground objects onto the location and movements of the hands in signing space. In addition, the retrieval of whole entity object classifiers recruits left IFG and left inferior temporal cortex (Emmorey et al., 2013).

Little is known about the neural regions that support sentence and phrase production in sign languages. The aphasia literature clearly indicates that the left hemisphere is critical for sentence production (Poizner et al., 1987; Atkinson et al., 2005), but the within-hemisphere functional organization is largely unknown. Clues from the early cases of sign language aphasia suggest that the left inferior parietal lobule may be involved in maintaining associations between spatial locations and referents, as well as directing verbs or pronouns toward these locations (Poizner et al., 1987; Corina et al., 1992). The MEG study by Blanco-Elorrieta et al. (2018) indicated that the left ATL and vmPFC are involved in phrase-level compositional processes, but the potential roles of left posterior temporal cortex or left IFG in sentence production are less clear. In contrast, more is known about the contribution of these regions to sentence comprehension in sign languages (see below).

Perhaps surprisingly, the sketch of the sign language production network in Figure 1 does not include left middle or posterior superior temporal cortex (STC). While many studies report activation in these STC regions for sign comprehension (see below), most studies of overt and covert sign production do not find activation in STC (Corina et al., 2003; Emmorey et al., 2003; Emmorey et al., 2004; MacSweeney et al., 2008b; Pa et al., 2008; Hu et al., 2011; Emmorey et al., 2016). Nonetheless, a few studies have reported activation in left posterior STC for covert sign production (Kassubek et al., 2004; Buchsbaum et al., 2005). Other studies that report left posterior STC activation used production tasks that were linked to the perception of signs, such as sign repetition (San José Robertson et al., 2004; Li et al., 2014). Interestingly, the conjunction analysis of ASL verb generation and action naming by San José Robertson et al. (2004) did not report STC activation. In contrast, studies of speech production consistently report bilateral STC activation, due at least in part to the auditory feedback that accompanies speech, and some models propose that auditory targets for production are represented in middle and posterior STC (e.g., the DIVA model; Tourville & Guenther, 2011). Thus, it appears that STC may not be as intimately involved in language production for sign as it is for speech.

Overall, output differences between sign and speech result in greater left parietal (SPL and SMG) engagement for sign compared to speech production (likely due to differences in the nature of phonological units across the two modalities), and greater engagement of left superior temporal cortex for speaking than signing (likely due to auditory feedback and auditory targets for speech production). Both sign and word production engage the left inferior frontal cortex, which is likely involved in lexical selection and lexical-semantic processes for both language types. At the sentence level, phrase production in sign and speech engage the same regions (left ATL and vmPFC), but much more research on sentence production by signers is needed to determine the extent of similarities and differences across modalities (e.g., confirming whether or not left SMG is uniquely engaged for reference establishment and the production of agreeing verbs and pronouns) (Figure 1).

## THE NEUROBIOLOGY OF SIGN LANGUAGE COMPREHENSION

As for language production, psycholinguistic evidence points to many parallel processes for the comprehension of signed and spoken languages. For example, at the phonological level, both signers and speakers become tuned to the phonological units of their language, as evidenced by categorical perception effects (Palmer et al., 2012), and both sign and speech are segmented using the same form-based constraints (e.g., the Possible Word Constraint; Orfanidou et al., 2010). Sign and word recognition are automatic, as evidenced by Stroop effects (Bosworth et al., 2021), and are both influenced by lexical frequency (faster recognition times for high frequency signs/words; Carreiras et al., 2008) and phonological neighborhood density (slower recognition for signs/words with many form-similar neighbors; Caselli et al., 2021). At the sentence level, the mechanisms for processing pronominal referents are parallel (e.g., antecedent re-activation; Emmorey et al., 1991), and the implicit causality of verbs guides pronoun interpretation for both language types (Frederikson and Mayberry, 2021). Of course, for sign languages, linguistic information must be extracted from a visual signal expressed by the body, whereas an acoustic signal is the primary information source for spoken languages. In this section we explore whether and how modality differences in perception impact the neural underpinnings of sign comprehension. The review covers neural regions involved in the perception of sublexical units of signs (handshape, location, movement), lexical sign recognition, and sentence comprehension. In addition, we examine comprehension of typologically-unique properties of sign languages: non-manual features, iconic signs, fingerspelling, and classifier constructions. Parallel to the review of sign production, we end with a summary sketch of the neural network that supports sign language comprehension.

### Perception of Sublexical Phonological Structure

For spoken language, bilateral auditory regions in superior temporal cortex differentiate speech from non-speech stimuli (e.g., Binder et al., 2000) and do so very rapidly, as early as 100–150 ms (e.g., Shtyrov et al., 2000). Similarly, using MEG, Almeida et al. (2016) demonstrated that the earliest visual cortical responses (M100 and M130) exhibited specific modulations in deaf signers to ASL signs (still images) that violated anatomical constraints (e.g., a left hand on a right arm); this early visual response was not observed for hearing non-signers. Deaf signers also exhibited increased perceptual sensitivity compared to non-signers (i.e., better discrimination between possible and impossible signs). These results indicate that the early neural tuning that underlies the discrimination of language from non-language information occurs for both speakers and signers, but in different cortical regions (superior temporal cortex for speech vs occipital cortex for sign). Further, visual cortex shows entrainment to visual oscillations (rhythms) in signing, and entrainment in visual cortex is not observed for non-signers watching ASL (Brookshire et al., 2017). Thus, although entrainment may be driven in part by low-level visual features of signing (e.g., quasi-periodic fluctuations in visual movements), it is also modulated by top-down sensory predictions based on linguistic knowledge.

Cardin et al. (2013) and Cardin et al. (2016) used functional magnetic resonance imaging (fMRI) and the sign language equivalent of a phoneme monitoring task to investigate the neural networks that support processing of handshape and location parameters. Participants (deaf BSL signers and deaf/ hearing non-signers) pressed a button when they detected a sign form containing a cued location or handshape. The stimuli consisted of BSL signs, pseudosigns, and illegal sign forms that violated phonological constraints (e.g., non-occurring handshapes or points of contact or illegal phonological combinations). For all groups and stimulus types, monitoring for handshape engaged regions involved in the representation of the hand/arm and hand/arm movement goals (bilateral intraparietal sulcus, inferior temporal cortex), while monitoring for location engaged regions involved in spatial attention and the localization of body parts (bilateral precuneus, angular gyrus, and medial prefrontal cortex). These findings indicate that perception and recognition of handshape and body locations are supported by distinct neural regions, but these networks are not modulated by linguistic knowledge (or structure). Similarly, sign movement, the third major phonological parameter in sign language, activates neural regions that are sensitive to biological motion (area MT+ in the posterior temporal lobe) in both signers and nonsigners (e.g., Levänen et al., 2001).

Cardin et al. (2013) and Cardin et al. (2016) also reported two cortical areas that were activated only in the deaf signing group, indicating that these regions were specifically engaged in linguistic processing: superior temporal cortex (STC) and SMG in both hemispheres. All stimuli (real signs, pseudosigns, and illegal signs) engaged bilateral STC to a greater extent for deaf signers than non-signers (deaf or hearing). This result suggests that language-like stimuli engage bilateral STC for signers, regardless of semantic content (or phonological structure). However, the precise role of left STC in processing the linguistic form of signs remains to be determined. Illegal sign forms that violated BSL phonotactic constraints elicited stronger activation in bilateral SMG only in deaf signers. One interpretation of this result is that bilateral SMG plays a role in the integration of phonological parameters during sign perception, such that phonological violations increase neural activity in this region, perhaps due to the difficulty of integrating non-occurring handshapes, locations, and combinations.

Electrophysiological evidence also indicates that handshape and location parameters are processed differently in the brain, but this difference is not purely perceptual and can impact lexical-level processes. Using an ERP priming paradigm and delayed lexical decision, Gutiérrez et al. (2012) found that location-only overlap between a prime and target sign led to a “reversed” N400 response (greater negativity for location-related than unrelated target signs) for Spanish Sign Language (LSE). The N400 is an ERP component associated with lexical-level processing (see Kutas and Federmeier, 2011, for review). Crucially, modulation of the N400 was not observed for pseudosign targets, indicating that location overlap impacted lexical, rather than sublexical processing. Handshape overlap did not modulate the N400 component, although a later (600–800 ms) more typical priming effect was observed (i.e., reduced negativity for handshape-related than unrelated targets). Gutiérrez et al. (2012) hypothesized that the increased N400 negativity for signs sharing location reflects lexical competition via lateral inhibition. Meade et al. (2021a) reported similar results for ASL and provided further support for this hypothesis. Deaf ASL signers performed either a go/no-go repetition detection task which could be performed based only on perceptual processing (lexical access/selection is not required) and a go/no-go semantic categorization task (is this a country sign?) which required lexical selection and semantic processing. Handshape-related targets elicited smaller N400s than unrelated targets (indicative of facilitation), but only for the repetition detection task. The N400 effect for location-related targets reversed direction across tasks: a smaller N400 amplitude for the repetition detection task (indicating facilitation), but a larger N400 in the semantic task, indicative of lexical competition. Together, these results provide evidence that handshape and location play different roles during sign recognition. Specifically, both handshape- and location-related prime signs can pre-activate sublexical representations of handshapes and locations and thus facilitate processing of target signs. However, at the lexical level, signs compete for selection (via lateral inhibition) and this competition appears to be primarily driven by the location parameter (see also Carreiras et al., 2008). Sublexical facilitation and lexical competition are features of interactive-activation models proposed for word recognition (e.g., McClelland and Elman, 1986), and these data indicate that such models and their neural underpinnings apply to sign recognition as well.

### Perception of Non-manual Features

Non-manual features (e.g., facial expressions, headshake, eye gaze) constitute a non-trivial component of the structure of sign languages. At the phonological level, non-manual features can distinguish between minimal pairs, such as the ASL signs NOT-YET and LATE, which are distinguished only by tongue protrusion produced with NOT-YET. At the syntactic level, distinct facial expressions (e.g., furrowed or raised eye brows) mark different types of questions, and headshake marks negation in many sign languages, although the scope of negation (when and where the headshake must occur) varies cross-linguistically (Zeshan, 2006). Eye-gaze has deictic, referential functions in many sign languages (Garcia and Sallandre, 2020). Despite the importance of non-manual features, very little is known about how they are represented and processed in the brain.

Two fMRI studies have examined how non-manual components of signs are processed in the brain. Capek et al. (2008) compared neural activity for comprehending BSL signs produced with and without non-manual features. Signs with non-manual features were produced either with mouthings (speech-derived mouth movements) or mouth gestures (mouth actions unrelated to speech). In this study, mouthings disambiguated manual BSL signs, e.g., mouthing “Asian” vs “blue” distinguishes the meaning of identical manual signs, representing a minimal pair distinguished only by mouthing. Mouth gestures were obligatory mouth actions that constituted a sublexical non-manual feature of the sign, e.g. producing a closing mouth gesture simultaneously with the downward movement of the hand in the sign TRUE (an example of “echo phonology” in which the mouth and hand movements resemble each other; Woll, 2014). Signs with non-manual features (both mouthing and mouth gestures) generated greater neural activity in the superior temporal sulcus (STS) in both hemispheres (extending into SMG in the left hemisphere) and in left IFG, compared to manual-only signs. This result suggests that bilateral STS and left IFG are involved in recognizing mouth actions that accompany manual signs, possibly because these regions are particularly sensitive to movements of the mouth, even for non-linguistic mouth articulations (Pelphrey et al., 2005). The fact that activation extended into left SMG suggests that this region may be involved in integrating both manual and non-manual phonological features during sign recognition. The contrast between signs with mouthing and those with mouth gestures revealed greater neural activity in left middle STS for signs with mouthing, and this region overlapped with the STS region activated when the same deaf BSL signers comprehended silent speech (Capek et al., 2010). Comprehending signs with mouth gestures generated greater neural activity in a more posterior region along the STS bilaterally, which overlapped with the posterior temporal regions engaged by manual-only signs. These findings suggest that mouthings may represent a form of language mixing (code-blending) since the recognition of mouthings was supported by the same left STS region engaged during speech-reading. In contrast, mouth gestures engaged the same bilateral posterior temporal regions involved in perceiving hand movements, possibly because their articulation is linked to the dynamic movements of the hands (Woll, 2014).

McCullough et al. (2005) investigated the neural underpinnings of ASL facial expressions that convey adverbial distinctions, such as “effortlessly” (lips pressed together) or “carelessly” (tongue protrudes). Deaf signers and hearing non-signers made same-different judgements to pairs of static images of different signers producing the same verb with either the same or a different non-manual adverbial; the baseline comparison was a same-different gender decision for images of signers producing verbs with neutral facial expressions. Neural activity for recognizing linguistic facial expressions (compared to the baseline) was strongly left-lateralized in posterior STS only for the deaf signers. The left STS neural activity for ASL adverbial facial expressions was posterior to the left STS region that Capek et al. (2008) reported for mouthings in BSL, but similar to the posterior STS region engaged for BSL mouth gestures. In addition, Capek et al. (2008) reported greater activation for mouth gestures than mouthings in the left fusiform gyrus, and McCullough et al. (2005) also found left-lateralized activity for adverbial facial expressions (compared to neutral faces) in the fusiform gyrus for deaf signers only. This region includes the fusiform face area, which is specialized for face perception (Kanwisher, 2000). Together these results indicate that comprehension of sublexical facial components of signs engages face-sensitive neural regions (posterior STS and the fusiform gyrus; Ishai, 2008). Furthermore, the finding that neural activity is larger in the left hemisphere indicates that these face-sensitive regions are modulated by linguistic processing demands and form part of the language network for sign language.

Finally, Atkinson et al. (2004), found that comprehension of non-manual negation in BSL (headshake with furrowed brows, narrowed eyes and/or downturned mouth) was impaired for signers with right hemisphere damage and spared for signers with left hemisphere damage. Atkinson et al. (2004) hypothesized that non-manual negation may function prosodically, rather than syntactically, under an analysis in which non-manual negation is associated with syntactic structure, but is not itself syntactic. This hypothesis fits with evidence that the right hemisphere is involved in prosodic processing for spoken language (e.g., Meyer et al., 2003), and with other linguistic evidence suggesting that non-manual negation can be a prosodic marker in sign languages (Pfau, 2008).

### Lexical Comprehension

A MEG study by Leonard et al. (2012) demonstrated that the initial neural response to lexical signs (80–120 ms post onset) occurs in bilateral visual (occipital) cortex, in contrast to the early neural response in auditory cortex for spoken words. In this study, deaf ASL signers performed a picture–sign matching task, while hearing English speakers performed a picture–auditory word matching task with the same items. Neither the early visual response to signs nor the early auditory response to words was sensitive to semantics (i.e., whether the sign was congruent or incongruent with the preceding picture). However, a later time window (300–350 ms) showed evidence of semantic sensitivity (greater negativities for incongruent than congruent trials) and high overlap in the neural regions for sign and word comprehension. Regions in bilateral STC (planum temporale, superior temporal sulcus, temporal pole) exhibited lexical semantic sensitivity, with a stronger response in the left than right hemisphere for both signs and words. However, the left intraparietal sulcus (IPS) exhibited semantic sensitivity only for ASL signs, suggesting a modality-specific role for this parietal region in the lexical semantic processing of signs. This finding points to the possible overlap in lexical processing for sign comprehension and production in left parietal cortex.

Emmorey et al. (2015) also observed left IPS activation when deaf ASL signers made semantic decisions to signs (concrete or abstract meaning?), in comparison to a low-level baseline task, and activation was not observed for hearing non-signers viewing the same ASL signs. Importantly, left IPS was not engaged when ASL signers made the same semantic decision to fingerspelled words, and the direct contrast between ASL signs and fingerspelled words revealed greater activation for ASL signs in left SMG (extending into IPS). ASL fingerspelling is produced at a single location in signing space and most fingerspelled letters are not specified for movement. Thus, to comprehend fingerspelled words, handshapes do not need to be integrated with locations on the body or with different movement types. In contrast, to recognize and comprehend ASL signs, all three phonological parameters must be integrated. In addition, ASL signs have stored lexical representations, whereas the fingerspelled words presented in the Emmorey et al. (2015) study did not; they were not loan signs and did not have ASL translations. The finding that neural activity in SMG is strongly left-lateralized when signers perform a lexical semantic task, but not when they perform a form-based monitoring task as found by Cardin et al. (2016), suggests that left SMG supports lexical-level phonological processing during comprehension. Right SMG may engage in form-based/phonetic-level processing of signs, particularly when lexical-semantic processing is not required.

Neuroimaging studies targeting lexical-level processing in deaf signers of several different languages consistently report bilateral activation in posterior STC, typically with more extensive activation in the left hemisphere: ASL (Corina et al., 2007; Emmorey et al., 2015); BSL (MacSweeney et al., 2006; Capek et al., 2008), CSL (Li et al., 2014), German Sign Language (Deutsche Gebärdensprache or DGS; Klann et al., 2002; Trumpp and Kiefer, 2018); Langue des Signes Québecoise (LSQ; Petitto et al., 2000); and Polish Sign Language (Polski Język Migowy or PJM; Banaszkiewicz et al., 2020). Interestingly, Inubushi and Sakai (2013) did not report activation in STC when deaf signers of Japanese Sign Language (JSL) performed a pseudosign detection task with unrelated sentences, but the baseline comparison was backward videos of sentences, and many signs remain intelligible when viewed backward, unlike reversed speech (Bosworth et al., 2020). Thus, because both the experimental and baseline tasks engaged lexical-semantic processing, activation in STC may have been cancelled out. Lesion data indicate that left STC (and likely left middle temporal gyrus) is critical to lexical semantic processing. ASL signers with damage to left posterior temporal cortex were more impaired in a sign-picture matching task than signers with cortical damage that spared this region (Hickok et al., 2002).

The neuroimaging studies cited above that investigated lexical comprehension in ASL, BSL, CSL, DGS, LSQ, and PJM all report additional neural activity within left inferior frontal cortex (IFC), often accompanied by less extensive neural activity in the homologous region in the right hemisphere. Left IFC includes Broca’s area (BA 44 and 45) along with a more anterior frontal region (BA 47); these regions have been associated with multiple functions for spoken word comprehension, including selecting among competing possible words, linking semantics with phonology, and maintaining words in memory (Hagoort, 2005; Price, 2012). Bilateral IFC has also been shown to be part of a fronto-parietal network for encoding and retrieving signs in working memory (Rönnberg et al., 2004; Bavelier et al., 2008). However, the precise role(s) of left IFC (and its subregions) and the homologous region in the right hemisphere in lexical-level comprehension for sign language requires further research.

#### Iconicity and Lexical Comprehension

Most of the evidence to date indicates that iconic signs are not comprehended differently in the brain compared to non-iconic signs. Atkinson et al. (2005) found that deaf signers with aphasia were equally impaired in their comprehension of iconic and non-iconic signs and did not use iconicity as a cue to sign meaning, in contrast to hearing non-signers performing the same sign-picture matching task. Using event-related fMRI and representational similarity analyses, Evans et al. (2019) found that iconicity did not influence the neural representation of BSL signs in left posterior middle/inferior temporal cortex. Emmorey et al. (2020) found no ERP effects of iconicity in a go/no-go semantic categorization task with deaf ASL signers, even though hallmarks of lexical access–frequency and concreteness effects–were observed during the N400 time window. Mott et al. (2020) also found no effects of iconicity during this time window for deaf signers in a cross-language translation recognition task, although effects of iconicity were found for hearing adult ASL learners.

Finally, a recent ERP study by McGarry et al. (2021c) examined the neural response when ASL signers performed a picture-sign matching task in which the picture either visually-aligned with the iconic sign (e.g., a bird in profile for the sign BIRD in which the fingers depict a bird’s beak) or was not aligned with the sign (e.g., a picture of a bird in flight). Replicating previous behavioral studies (Thompson et al., 2009; Vinson et al., 2015), signers were faster for the aligned than non-aligned trials. However, the ERP data indicated that aligned trials were associated with a reduced P3 amplitude rather than a reduced N400, suggesting that visual picture-sign alignment facilitated the decision process (indexed by the P3 component), rather than lexical access (indexed by the N400 component). Thus, the faster response times found in these studies for the picture-aligned trials likely occurred because it was easier for participants to make the picture-sign matching decision, rather than to a priming effect in which the visual alignment between the picture and the iconic sign facilitated sign recognition and lexical access.

#### Comprehension of Fingerspelled Words

Two studies have examined the neural underpinnings for the comprehension of fingerspelled words, investigating the two-handed BSL system (Waters et al., 2007) and the one-handed ASL system (Emmorey et al., 2015). The contrast between fingerspelled words and lexical signs for both ASL and BSL revealed greater neural activity for fingerspelling in the visual word form area (VWFA) located in ventral occipito-temporal cortex. Differential VWFA activation was not found for hearing sign-naïve controls, indicating that linguistic knowledge underlies this result and that it cannot be accounted for by perceptual differences between fingerspelling and signing. Waters et al. (2007) also found that the right VWFA was engaged by fingerspelled words, which could reflect the fact that skilled deaf readers tend to engage the VWFA bilaterally for written words (Glezer et al., 2018; see also Emmorey et al., 2017). Overall, both studies found that fingerspelling was more left-lateralized than signing, which parallels the finding that reading text is more left-lateralized than listening to speech (Buchweitz et al., 2009). Like text, fingerspelling is acquired later in childhood when children learn to make associations between handshapes and written letters, and both reading and fingerspelling build on already established left-hemisphere language regions. In sum, both the comprehension and production of fingerspelled words recruit the VWFA, highlighting its role in accessing orthographically structured representations.

### Sentence Comprehension

Neuroimaging studies of sentence-level comprehension in different sign languages report engagement of a similar bilateral fronto-temporal network as found for single sign comprehension, again with more extensive activation in the left hemisphere: ASL (Newman et al., 2010; Mayberry et al., 2011), BSL (Mac Sweeney et al., 2002a; MacSweeney et al., 2006), CSL (Liu et al., 2017), DGS (Gizewski et al., 2005), JSL (Sakai et al., 2005; Inubushi & Sakai, 2013), Langue des Signes Francaise (LSF; Moreno et al., 2018), and PJM (Jednoróg et al., 2015). Lesion data from ASL signers support the hypothesis that left posterior temporal cortex is critical to sentence comprehension (Hickok et al., 2002). For example, on simple sentences from the Token Test involving single clause commands (e.g., “point to any square”), signers with left posterior temporal damage (extending into parietal cortex) scored 53% correct, while signers with left hemisphere lesions that did not involve temporal cortex scored near ceiling. A few neuroimaging studies have worked to isolate the neural regions that are specifically engaged in sentence-level comprehension processes. MacSweeney et al. (2006) found that BSL sentences engaged left IFG and left posterior temporal cortex to a greater extent than lists of BSL signs, indicating that this left fronto-temporal network is recruited when words are integrated to create meaning.

Inubushi and Sakai. (2013) probed word-, sentence-, and discourse-level processing by presenting JSL sentences to deaf signers who performed a pseudoword detection task or a grammatical error detection task (e.g., verb agreement or word order violations) for sets of unrelated sentences. For the discourse-level task the same sentences constituted a dialogue between two signers, and the task was to detect a sentence that did not fit into the conversation, but was otherwise syntactically and semantically well-formed. The baseline task was to detect a repeated video from the same sentences played backwards. A key result of this study was that as attention shifted to higher levels of processing, neural activity increased in the following left IFG regions (word < sentence < discourse): lateral premotor cortex (LPMC), Broca’s area (BA 44, 45), and anterior IFG (BA 47). Furthermore, neural activity expanded along this dorsal-ventral axis as attention shifted to higher linguistic levels, moving from words (left LMPC) to sentences (left BA 44, 45) to discourse (bilateral BA 47).

The role of the left IFG in sign language comprehension was further documented in a recent ALE (activation likelihood estimate) meta-analysis by Trettenbrein et al. (2021) of 23 fMRI/PET studies with deaf signers (*N* = 316) of seven different languages. The meta-analysis revealed bilateral IFG activation extending into lateral premotor cortex in the left hemisphere for sign comprehension compared to control/baseline tasks (more than half of the contrasts involved sentence processing). Activation in Broca’s area (BA 44, 45) was strongly left-lateralized, particularly for BA 44. Further, a comparison with another meta-analysis of the perception of non-linguistic action gestures (by non-signers) indicated that sign language comprehension engaged left IFG (with a peak in BA 44) to a greater extent than human action observation, but the conjunction analysis revealed overlap in right IFG (BA 45). Based on these results, Trettenbrein et al. (2021) hypothesized that left IFG computes linguistic aspects of sign language during comprehension, while right IFG may be involved in processing aspects of the visual-manual signal that are shared with action gestures. Similarly, a graph theoretical analysis of fMRI data from hearing signers comprehending CSL sentences compared to non-signers observing these sentences revealed that left BA 44 served as a central network hub in signers only (Liu et al., 2017), supporting the hypothesis that this region plays a role in integrating information within the language network for sign language.

A typologically unique feature of sign language syntax is the use of “agreeing” verbs that can be directed toward locations in signing space to express grammatical relations (see Mathur and Rathmann, 2012, for an overview). In these sentence types, referents are associated with locations in signing space and the agreeing verb can be directed toward these locations to indicate grammatical roles (e.g., subject, object). Electrophysiological and neuroimaging data suggest that comprehension of sentences with these verbs may engage spatial processing mechanisms that are unique to the signed modality. Similar to ERP results from spoken languages, Capek et al. (2009) observed an early anterior negativity followed by a P600 response to syntactic violations involving ASL agreeing verbs. However, the distribution of the anterior negativity differed depending upon the type of agreement violation. Violations in which the verb direction was reversed (moving toward the subject instead of the object location) elicited a left anterior negativity, whereas for violations in which the verb was directed toward a previously unspecified location, the anterior negativity was larger over the right hemisphere.

Following up on this study, Stroh et al. (2019) used fMRI to probe the underlying neural correlates of processing verb agreement violations. DGS signers were presented with sentences that contained an agreement violation in which the incorrect direction of movement was from neutral space to the first person, as well as sentences that contained a semantic violation and correct sentences (task: acceptability judgment). Sentences with semantic violations vs correct sentences elicited greater activation in left calcarine sulcus (a low-level visual processing region) and left IFG. Stroh et al. (2019) hypothesized that the increased neural activity in early visual cortex reflected sensitivity to violations of sensory predictions of the incoming signed signal (the semantically anomalous word always occurred at the end of the sentence), whereas left IFG activation likely reflected lexical semantic integration processes. Syntactic violations elicited greater neural activity in right SMG compared to both correct sentences and compared to sentences with semantic violations; left IFG did not exhibit sensitivity to agreement violations. Stroh et al. hypothesized that right SMG is involved in attending to and tracking the spatial location of referents. Together these results confirm the role of left IFG in sentence-level semantic processes and point to the role of the right SMG in comprehending “spatial syntax” expressed by agreeing verbs.

An fMRI study of ASL signers by Newman et al. (2010) also points to the possible role of bilateral superior temporal cortex (STC) in comprehending sentences with agreeing verbs and other types of simultaneous morphology (e.g., aspectual inflections and numeral incorporation). This study compared the comprehension of ASL sentences that contained signs inflected with grammatical morphology with sentences that contained the same uninflected signs or lexical signs that conveyed the same information (e.g., lexical adverbs or separate number signs). The sentences that contained simultaneous morphology elicited greater activation in anterior and posterior STC bilaterally and in a region in left IFG (BA 45) compared to sentences without morphology. This pattern resembles the fronto-temporal network hypothesized to support the recognition and interpretation of inflectional morphology in spoken languages (Marslen-Wilson and Tyler, 2007).

Finally, Matchin et al. (2021), recently investigated the neural regions involved in syntactic/semantic combinatorial processes by presenting ASL signers with stimuli of the same length, but which parametrically increased in the size of the linguistic constituents: lists of six unrelated signs, three two-sign simple sentences (e.g., subject verb), and complex six-sign sentences (e.g., sentences with embedded clauses). The neural region that was sensitive to this parametric variation in combinatorial structure was the left STS with an anterior and a posterior peak of activation. This result mirrors what has been found for spoken language (French) using a very similar paradigm (Pallier et al., 2011). In addition, the anterior STS peak was in the left ATL, suggesting that this region supports combinatorial processes for both comprehension and production. Interestingly, left IFG was not sensitive to the manipulation of syntactic combinatorial structure in ASL. This result is consistent with other studies that have failed to find evidence that Broca’s area (BA 44, 45) is a core region involved in syntactic structure building during comprehension of spoken languages (e.g., Brennan and Pylkkänen, 2012). Recently, Matchin and Hickok. (2020) argued that the role of Broca’s area in syntactic processing is primarily tied to production (linearalizing lexical information from posterior temporal cortex) and that during comprehension activation in Broca’s area is driven by working memory resources.

#### Comprehension of Classifier Constructions

As with production, results from lesion studies indicate that either left or right hemisphere damage can result in comprehension deficits for classifier constructions expressing spatial relationships (Emmorey et al., 1995; Atkinson et al., 2005). However, the neuroimaging evidence for right hemisphere involvement is somewhat mixed. Using fMRI and a semantic anomaly detection task, MacSweeney et al. (2002b) compared neural activation for deaf BSL signers when comprehending topographic versus non-topographic sentences. Topographic sentences used signing space and/or the signer’s body to express spatial information, while non-topographic sentences did not. The contrast between these two sentence types revealed greater activation for the topographic sentences in left but not right parietal cortex. However, the topographic sentences did not focus specifically on spatial relationships and included a wide range of constructions, e.g., *The woman shaved her legs* and *I flew from London to Dublin* (English translations). It is possible that activation in right parietal cortex was not observed for the topographic sentences because only a handful of sentences required mapping the location of the hands in signing space to the location of figure and ground referents (e.g., *The cat sat on the bed*).

Using event-related fMRI and a sentence-picture matching task, a recent study by Emmorey et al. (2021) specifically targeted the comprehension of ASL locative classifier constructions by deaf signers and their English translations by hearing speakers. The sentences expressed either a perspective-dependent spatial relationship (*left, right, in front of, behind*) or a perspective-independent relationship (*in, on, above, below*) between a figure and ground object. In contrast to non-spatial control sentences, perspective-dependent sentences engaged SPL bilaterally for both ASL and English, consistent with a previous study using the same design with written English (Conder et al., 2017). The ASL-English conjunction analysis revealed bilateral SPL activation for perspective-dependent sentences, but left-lateralized activation for perspective independent sentences. The direct contrast between perspective-dependent and perspective-independent expressions revealed greater SPL activation for perspective-dependent expressions only for ASL. Emmorey et al. (2021) hypothesized that the increased SPL activation for ASL perspective-dependent expressions reflects the mental transformation required to interpret locations in signing space from the signer’s viewpoint (Brozdowski et al., 2019).

Newman et al. (2015) failed to find activation in either left or right parietal cortex when deaf ASL signers comprehended sentences containing location and motion classifier constructions (descriptions of animations designed specifically to elicit these constructions; Supalla, 1982). These sentences were compared to a “backward/layered” control condition in which the sentence videos were played backward with three different videos superimposed. The experimental task was to decide whether a sentence matched a preceding video, and the control task was to determine whether three hands had the same simultaneous handshape in the backward/layered video. Compared to this control condition, comprehension of ASL location and motion classifier constructions engaged bilateral STC and left IFG, i.e., the neural network described above for sentence comprehension in sign languages. However, the visually complex baseline video and spatial attention task may have swamped any additional parietal activation related to interpreting expressions of motion or location in the signed sentences. When neural activity for the ASL sentences was compared to a fixation baseline, neural activity in both left and right parietal cortices was found (Supplementary Table S1 in Newman et al., 2015).

MacSweeney et al. (2002b) also found that a motion-sensitive region, area MT+, in bilateral posterior temporal cortex was more engaged for BSL topographic sentences (some of which expressed the movement of a referent) compared non-topographic sentences. McCullough et al. (2012) followed up on this result and targeted the comprehension of ASL sentences with classifier constructions that expressed motion (e.g., *The deer walked along a hillside*) versus matched sentences that expressed static location information (e.g., *The deer slept along a hillside*). MT+ was localized individually for each deaf signer using motion flow fields. The results revealed greater neural activity in bilateral MT+ for motion compared to location sentences. This finding indicates that linguistic semantics modulates motion-sensitive cortex (cf. Saygin et al., 2010). Further, this top-down modulation is not disrupted by the visual motion in the signed signal, possibly because the physical movement of the hands and the motion semantics are always congruent (e.g., an upward hand movement expresses upward motion of a referent). In addition, locative sentences engaged left parietal cortex to a greater extent than motion sentences, consistent with the left parietal activity observed when producing locative classifier constructions.

Finally, Jednoróg et al. (2015) compared comprehension of PJM sentences that expressed the same location and movement concepts either with classifier constructions or with lexical signs (passive viewing by deaf signers). Bilateral SMG and right SPL were more active during the comprehension of sentences with classifier constructions than sentences without them. The reverse contrast revealed greater activation in anterior STC bilaterally (more extensive on the left) for the sentences with lexical signs only. These differences were not observed when sign-naïve participants viewed the PJM sentences. Right SPL and bilateral SMG activation for comprehending classifier constructions is consistent with the lesion data and neuroimaging results from production studies. The greater activity for lexical sentences in left anterior STC extended into the left ATL, and Jednoróg et al. (2015) hypothesized that this region may be involved in semantic combinatory processes for lexical signs. This finding and interpretation are consistent with the left anterior STC region that Matchin et al. (2021) found to be sensitive to combinatorial structure in ASL and that Blanco-Elorietta et al., 2018) found to be engaged in phrase production.

### Sign Language Comprehension: Summary

**FIGURE 2 |:**
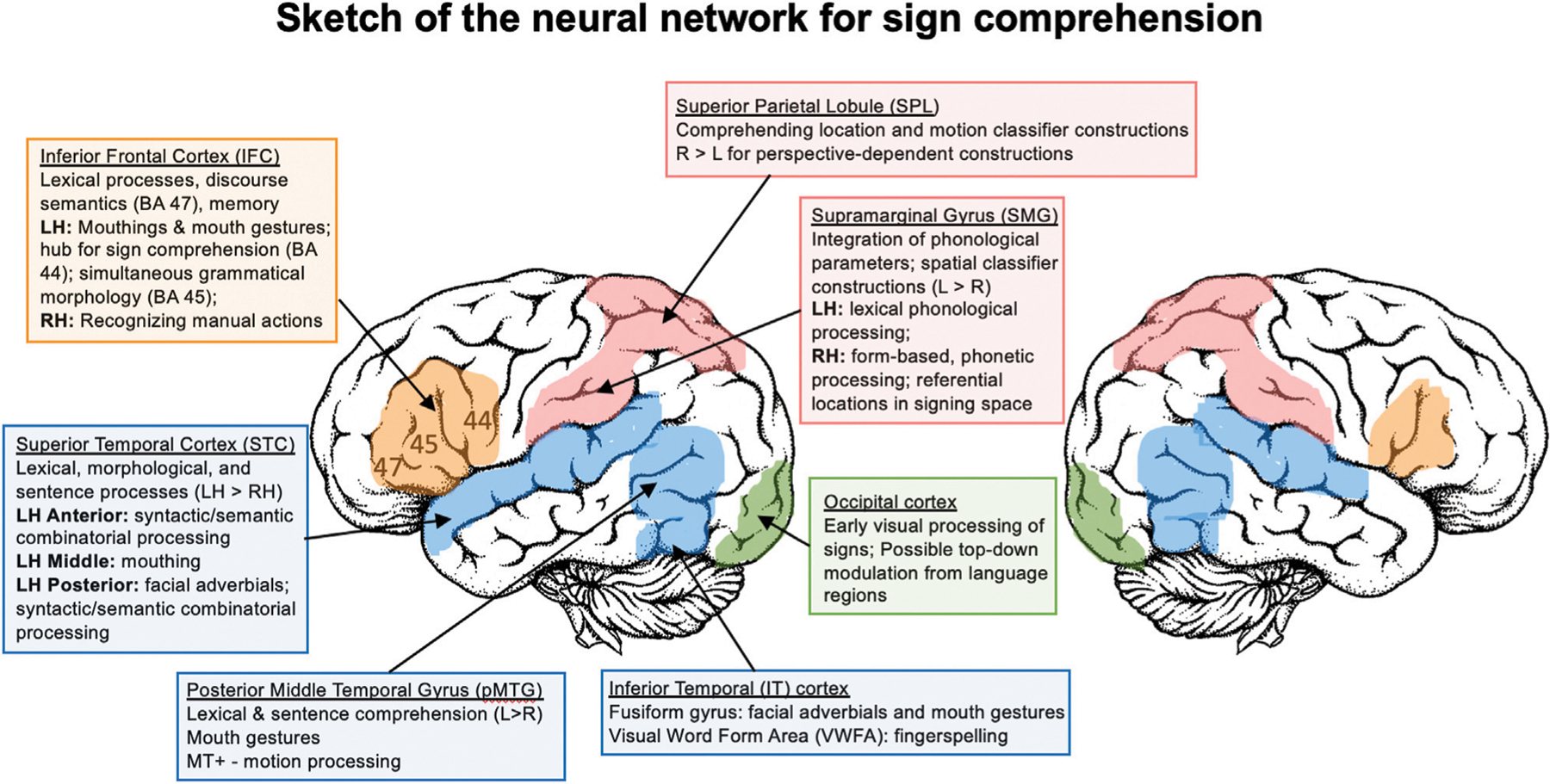
A sketch of the neural network that supports the comprehension of sign language. LH = left hemisphere; RH = right hemisphere.

Figure 2 provides a sketch of the neural network for sign language comprehension based on the studies reviewed here. With respect to the early phonetic/phonological perception of signs, occipital cortex appears to be tuned to quickly discriminate linguistic from non-linguistic stimuli in deaf signers (Almeida et al., 2016; see also Corina et al., 2007), and activation in early visual cortex can be modulated by top-down linguistic processes (Brookshire et al., 2017). For both signers and non-signers, neural regions involved in the perception of hand movements and body locations are recruited during sublexical processing of sign stimuli (i.e., detecting a specific handshape or body location) (Cardin et al., 2016). Bilateral STS is more activated when signers (vs non-signers) engage in sublexical processing, but this activation is not influenced by lexicality or phonological structure; thus, the precise role of STS in the sublexical processing of signs is unclear (Cardin et al., 2013). Bilateral SMG may be involved in the integration of phonological parameters during sign perception, as only signers exhibited sensitivity in SMG to phonological violations (Cardin et al., 2016). The electrophysiological data indicate that signers develop neural sublexical representations of handshapes and locations that can be primed (Meade et al., 2021a; see also Meade et al., Forthcoming). In addition, lateral inhibition between signs sharing location results in an increased N400 response when lexical selection is required to perform the task (Gutierrez et al., 2012; Meade et al., 2021a). These distinct electrophysiological responses provide evidence for a hierarchical organization of sublexical and lexical representations in the brain for sign languages.

There is some suggestion that left SMG may also be involved in integrating mouth actions with manual signs during sign recognition (along with left IFG) (Capek et al., 2008). Recognition of mouthings appears to rely on the same left middle STS region that supports speech-reading, while mouth gestures and adverbial facial expressions engage more posterior regions of STS. Comprehension of both mouth gestures and facial adverbials also engages the left fusiform face area in inferior temporal cortex (McCullough et al., 2005; Capek et al., 2008). Thus, the temporo-parietal network involved in phonological decoding of the manual and non-manual features of signs includes bilateral SMG and STS (more extensive in the left) and the left fusiform gyrus. However, comprehension of non-manual negation may be right-lateralized because right, not left hemisphere damage impairs its comprehension (Atkinson et al., 2004).

Comprehending lexical signs engages bilateral inferior frontal, posterior superior temporal, and inferior parietal cortices (e.g., MacSweeney et al., 2006; Emmorey et al., 2015). Lexical-semantic processing is associated with neural activity in IFC and posterior STC bilaterally, and lesion studies suggest that left posterior STC is critical for lexical sign comprehension (Hickok et al., 2002). Left inferior parietal cortex (SMG extending into IPS) may be involved in phono-lexical processing during sign comprehension, interfacing between semantic and phonological processing (Leonard et al., 2012; Emmorey et al., 2015). The precise contributions of left IFC in comprehending lexical signs requires further investigation, but there is evidence that this region plays a role in maintaining signs in memory (see also Buchsbaum et al., 2005; Pa et al., 2008). Thus far, there is little evidence that iconicity modulates the neural response during sign comprehension (e.g., Emmorey et al., 2020). However, it should be noted that neither the subjective nature of iconicity (Occhino et al., 2017) nor the different types of iconic mappings (e.g., Caselli and Pyers, 2020) have been taken into account in these studies. Finally, comprehension of both one-handed and two-handed fingerspelled words activates the visual word form area in ventral occipito-temporal cortex (Waters et al., 2007; Emmorey et al., 2015).

Although most neuroimaging studies of signed sentence comprehension find bilateral fronto-temporal activation in comparison to low-level baselines, studies that specifically target sentence-level computational processes find left-lateralized activation in superior temporal cortex. In particular, posterior STC and the left ATL may be involved in syntactic/semantic integration processes for lexical signs within phrases or sentences (Matchin et al., 2021). Left IFG (particularly BA 44, 45) appears to be a hub in the sentence processing network for sign languages, as for spoken languages, with several possible integrating and memory functions (e.g., Bavelier et al., 2008; Inubushi and Sakai, 2013). Left IFG (BA 45) along with bilateral STS appears to support comprehension of simultaneous morphology in signed sentences (Newman et al., 2010), but right SMG may be recruited to track the direction of agreeing verbs and the location of referents in signing space (Stroh et al., 2019).

Neuroimaging studies targeting comprehension of sentences with classifier constructions indicate involvement of left or bilateral SMG (MacSweeney et al., 2002b; McCullough et al., 2012; Jednoróg et al., 2015; Emmorey et al., 2021). In addition, bilateral SPL is implicated in comprehending classifier constructions that express object locations, and SPL may be particularly involved when signers comprehend perspective-dependent expressions, due to the mental transformation required to interpret spatial locations from the signer’s perspective (Emmorey et al., 2021). In addition, motion sensitive regions in bilateral posterior temporal cortex are engaged when comprehending sentences with classifier constructions that express movement (McCullough et al., 2012), as found for spoken language (Saygin et al., 2010).

Overall, the input differences between sign and speech comprehension can be seen, not surprisingly, in the early involvement of occipital (visual) versus superior temporal (auditory) cortices (Leonard et al., 2012). Similar to sign production, parietal cortex (bilateral SMG) appears to be more involved in form-level processing of signs (Cardin et al., 2016), likely due to differences in phonological units (e.g., handshapes and body locations, rather than consonants and vowels). Comprehension of both signed and spoken language engages IFC bilaterally (left > right). Left IFC (particularly BA 44) may serve as central hub in the language network (Liu et al., 2017), while right IFC (BA 45) may be involved in modality-specific processing of human manual and body actions (Trettenbrein et al., 2021). Sentence comprehension for both signed and spoken language relies on left STC, and many studies have now demonstrated a parallel left-lateralized fronto-temporal network for sign and speech comprehension (e.g., Mac Sweeney et al., 2002a; Sakai et al., 2005; Emmorey et al., 2014) (Figure 2).

## CONCLUSION AND FUTURE DIRECTIONS

In the 60 years since Stokoe’s linguistic description of ASL, there is abundant evidence for overlap in the neurobiology of spoken and signed languages, particularly around perisylvian cortex. However, much work remains to understand the specific neural computations that are involved in the production and comprehension of sign languages. Although a given neural region may be engaged for both signed and spoken language, it is possible that the neural computations within that region are not identical for the two modalities. For example, it is possible that posterior superior temporal cortex performs somewhat different computations during sign versus word comprehension. For sign languages, this region might primarily be engaged in accessing lexical semantic representations, while for spoken languages this region may be additionally engaged in mapping auditory-vocal phonological representations onto lexical semantic representations. Similarly, left inferior parietal cortex may be involved in phonological processes for both sign and speech, but may perform different functions due to modality differences in the nature of phonological units.

In fact, Evans et al. (2019) specifically investigated whether the lexical-semantic representations of signs and words overlapped within left posterior middle temporal gyrus (pMTG) in hearing native BSL-English bilinguals. The results revealed that although left pMTG was engaged during both sign and word comprehension, there was little evidence for a direct mapping between signs and words in this region, although neural overlap was demonstrated for the cross-linguistic representation of semantic categories (fruits, animals, transport) in left posterior middle/inferior temporal gyrus. Evans et al. (2019) speculated that the difference in the neural representations of words versus signs could be driven by differences in cascading activation from auditory-vocal versus visual-manual phonological forms. This review agrees with the conclusions of Evans et al. (2019) that we need to rethink the assumption of identical neural processes underlying sign and speech processing and “highlight the unique perspective that sign language can provide on language processing . . (p. 7).”

A comparison of the neural network for sign production (Figure 1) and comprehension (Figure 2) suggests that left IFG and left SMG are activated for both processes (see also Okada et al., 2016). The precise functions of this dorsal fronto-parietal circuit are unclear, but one possible role is to support lexical selection and the integration of phonological and semantic information. The posterior MTG is also engaged during both production and comprehension, and based on data from spoken language (Levelt and Indefrey, 2004), pMTG may be involved in conceptually-driven lexical retrieval and access, which is more left-lateralized for production and extends into left inferior temporal cortex (for picture-naming tasks). Also parallel for comprehension and production, the visual word form area (VWFA) supports the interface between fingerspelling and orthographic representations, but the nature of this interface requires more research. Superior parietal cortex is also involved in both sign production and comprehension, but likely performs different functions for production (e.g., planning and monitoring articulation) versus comprehension (e.g., spatial analysis of classifier constructions). Overall, the neural networks sketched in these two figures reveal that 1) the “classical model” of brain and language focused on Broca’s and Wernicke’s areas is woefully underspecified (just as it is for spoken language; Poeppel et al., 2012), and 2) the unique linguistic properties of visual-manual languages need to be accounted for in a neurobiological model of sign language.

In conclusion, there is clearly much work left to be done. The neural regions that support syntactic production in sign language are largely unknown, and this question is critical given that the nature of syntactic processing may differ for production and comprehension (Matchin and Hickok, 2020). Further, we still know very little about the timing of linguistic processes or the functional connections within the neural networks for sign language. For example, what is the time course of phonological assembly and decoding for sign production and comprehension? How are linguistically-relevant parietal cortices functionally connected with temporal and frontal cortices? The summary Figures 1 and 2 provided here contain no information about the temporal flow of linguistic information between regions or about how different regions function together. Future neurobiological models also need to account for the nature of the neural computations that are involved in sign language comprehension and production, just as researchers are building models that specify the neural computations for spoken language processes (e.g., Flinker et al., 2019).

There are also open domains of inquiry that are unique to sign languages. We know almost nothing about the neural regions that support the production of non-manual components at the phonological, lexical, or syntactic levels. In addition, almost all of the research reviewed here has been conducted with deaf native signers (i.e., those born into signing families), which constitute only 5–10% of deaf children (Mitchell and Karchmer, 2004). We know very little about how the early developmental experiences of deaf people born into hearing families impact the neural circuits for sign language processing, but see Mayberry et al. (2011), MacSweeney et al. (2008b), and Twomey et al. (2020) for some data on this question. We also know very little about the neural development of systems that support sign language and whether the developmental trajectory parallels that found for spoken language (but see Payne et al., 2019), particularly given the possible effects of early language deprivation on neural structure and function (Hall, 2017; Romeo et al., 2018; Meek, 2020).

In sum, it is hoped that over the next few decades we will enhance and deepen our understanding of the neurobiological foundations of sign language, which will not only provide further insights into the neural basis of human language, but will also provide a translational foundation for treating injury to the language system, for diagnosing language impairment in signers, and for promoting healthy brain development in deaf children.
